# Column-based removal of high concentration microplastics in synthetic wastewater using granular activated carbon

**DOI:** 10.1080/21655979.2023.2276391

**Published:** 2023-11-09

**Authors:** Nik nor Amirah Mohd Napi, Naimah Ibrahim, Muhammad Adli Hanif, Masitah Hasan, Farrah Aini Dahalan, Achmad Syafiuddin, Raj Boopathy

**Affiliations:** a Faculty of Civil Engineering and Technology, Universiti Malaysia Perlis, Arau, Malaysia; b Centre of Excellence for Water Research and Environmental Sustainability Growth (WAREG), Universiti Malaysia Perlis, Arau, Malaysia; c Environmental Health Division, Department of Public Health, Universitas Nahdlatul Ulama Surabaya, Surabaya, Indonesia; d Center for Environmental Health of Pesantren, Universitas Nahdlatul Ulama Surabaya, Surabaya, Indonesia; e Department of Biological Sciences, Nicholls State University, Thibodaux, LA, USA

**Keywords:** Plastic waste, microplastics, column-based removal, granular activated carbon, adsorption

## Abstract

Microplastic (MP) is an emerging contaminant of concern due to its abundance in the environment. Wastewater treatment plant (WWTP) can be considered as one of the main sources of microplastics in freshwater due to its inefficiency in the complete removal of small MPs. In this study, a column-based MP removal which could serve as a tertiary treatment in WWTPs is evaluated using granular activated carbon (GAC) as adsorbent/filter media, eliminating clogging problems commonly caused by powder form activated carbon (PAC). The GAC is characterized via N_2_ adsorption–desorption isotherm, field emission scanning electron microscopy, and contact angle measurement to determine the influence of its properties on MP removal efficiency. MPs (40–48 μm) removal up to 95.5% was observed with 0.2 g/L MP, which is the lowest concentration tested in this work, but still higher than commonly used MP concentration in other studies. The performance is reduced with further increase in MP concentration (up to 1.0 g/L), but increasing the GAC bed length from 7.5 to 17.5 cm could lead to better removal efficiencies. MP particles are immobilized by the GAC predominantly by filtration process by being entangled with small GAC particles/chips or stuck between the GAC particles. MPs are insignificantly removed by adsorption process through entrapment in GAC porous structure or attachment onto the GAC surface.

## Introduction

Microplastic (MP), an emerging contaminant that is present abundantly in the aquatic environment, has garnered a lot of interest because of the potential hazards and associated environmental issues due to its small size and affinity toward other contaminants [1]. Several studies have reported the capability of MPs in adsorbing various contaminants such as pesticides, antibiotics, and heavy metals such as cadmium and copper [2–5]. MPs are classified as plastic particles smaller than 5 mm and can be classified into two groups depending on their origin: (i) primary MPs, which consist of plastic particles specifically manufactured in micro-sizes and commonly utilized in personal care products e.g. toothpaste, scrubs, facial cleansers, etc. and (ii) secondary MPs, which are produced due to physical, chemical, or biological degradation of larger sized plastics [6]. In 2016, the reported amount of plastics in the aquatic environment was between 19 and 23 million metric ton and the amount was estimated to exponentially increase to 53 million metric ton in 2030 [7]. Previous studies have also specifically reported on the estimated amounts of MPs in the future based on modelling approaches. van Wijnen et al. [8] estimated that the amount of MP particles in 2050 could be doubled if enhanced waste management is not applied or instead, halved if applied. On the other hand, Everaert et al. [9] utilized meta-analysis using data available in the literature and reported that the amount of MP is expected to increase from 9.6 to 48.8 particles/m^3^ in 2100. The best course of action is to reduce the usage of plastics, especially those of single use; however, this initiative is only expected to produce a substantial impact in the long run. Therefore, significant efforts need to be taken to mitigate current MP pollution in the fresh and ocean waters.

Various studies reported that conventional wastewater treatment plants (WWTPs) are able to remove MP particles with removal efficiencies of greater than 90% [10]. Most WWTPs are not built for the removal of small MP particles; however, large MP particles can be practically eliminated at the primary (screening and grit chamber) and secondary (biological) treatment stages [11]. Nonetheless, due to their small and lightweight properties, smaller-sized MP particles (especially those below 100 μm in size) can be resistant to removal by conventional WWTP operations as these particles easily escape all treatment stages due to their miniscule size. Considering the huge influx of water treated daily in WWTPs, large amounts of MP particles are able to exit the effluent stream, thus making WWTP a consistent source of MP contamination in fresh water [12]. The amount of MP particles present in the effluent stream is strongly dependent on the size and operation of the WWTP and the amount of wastewater treated, which significantly influences the amount of MP present in the influent stream. Two WWTPs in Adana, Turkey, reported that their influent wastewater contained 1–6.5 million of MP particles daily, which were reduced to 220,000–1.5 million particles in the effluent [13]. Similarly, three WWTPs in South Carolina, USA, reported a discharge of 500–1000 million MP particles per day, which were reduced from 1000 to 20,000 million MP present in the effluent [14]. Additionally, MPs retained by the WWTPs are contained in the generated sludge, which would result in the release of MP particles in soil as the sludge is commonly processed and reused as agricultural fertilizer [15–17]. Proper MP removal method is desperately needed to reduce its release into the environment to complement or become an alternative treatment method to coagulation, flocculation. and sedimentation (CFS), filtration, and membrane bioreactor, which have been studied for MP removal with varying degrees of efficiency.

Column-based removal is an appealing and effective method in removing various pollutants e.g. heavy metals, dyes, pharmaceuticals, etc. from aquatic environments [18–20]. Various types of media have been utilized in column-based pollutant removals e.g. activated carbon, biochar, zeolites, molecular sieves, etc. The utilization of these materials provides additional benefits to the overall process due to their affordability and accessibility. Commercial products such as granular activated carbon (GAC) are highly preferred as the particle size can be controlled to enhance MP removal, address clogging issues, and facilitate regeneration process. Parameters such as pollutant concentration and bed length are essential in the design of columns/reactors to achieve the optimized column removal of pollutants [19,21,22]. Even though column-based removal is typically utilized for dissolved pollutants, various studies have reported the capability of this method in removing suspended solids present in the wastewater [23,24]. Based on this observation, MP particles which are commonly suspended in various aquatic environments theoretically can be eliminated via column-based removal.

Few studies have reported the removal of particles from aquatic environments via column-based methods with reported removal efficiencies between 83.5% and 100% [12,25–27]. The method consists of passing MP-containing water into columns filled with media, which acts to immobilize the MP particles, resulting in cleaner water in the effluent stream. MP particles may be immobilized by the media through two possible means: (i) by filtration as the MP particles are unable to pass through the media bed inside the column or (ii) by adsorption on the media surface through electrostatic attraction, hydrophobic interaction, and hydrogen bonding [12,28,29]. However, the majority of the studies reporting on MP column-based removal does not include studies on the effects of different MP concentrations and media bed lengths, which are essential for the design of the column to achieve high performance. In this study, the removal of MP-containing wastewater using a laboratory-scale glass column with GAC as the filter media/adsorbent was evaluated in a continuous-flow experiment. The properties of the GAC media e.g. porosity, surface morphology, and hydrophobicity were characterized and analyzed to determine their role in facilitating the GAC to remove MP particles. Two parameters, i.e. MP concentration and GAC bed length, were varied to investigate their influence on the MP removal efficiency by the column-based removal method.

## Materials and methods

### Materials

MPs (polyethylene, Sigma Aldrich) of particle size between 40 and 48 μm were employed as the model MP in this study and used as received. GAC (charcoal activated granular, Bendosen) utilized as the adsorbent/filter media were commercially purchased. The GAC particle size is between 1.16 and 2.5 mm based on particle size analysis. Ethanol (denatured, 99.9%, R&M Chemicals) was utilized in the preparation of synthetic MP-containing wastewater.

### Characterization of GAC

Surface area and porosity of the GAC were characterized via N_2_ adsorption–desorption isotherms (Tristar 3000) at 77.35 K, using Brunauer–Emmett–Teller (BET) and Barrett–Joyner–Halenda (BJH) model, respectively. The morphology of the GAC was identified using field emission scanning electron microscope (Zeiss Leo 1525) with type II secondary electron image (SE2) signal at magnification of ×5000.

The hydrophobicity/hydrophilicity of the GAC was determined using sessile droplet contact angle measurement [30]. The contact angle was determined using ImageJ software with contact angle plugins. The measurement was conducted at different points on the GAC surface, and the value reported is an average of three measurements. Based on the contact angle, the GAC is classified as follows: hydrophilic (θ < 90°), hydrophobic (90° < θ < 150°), and superhydrophobic (>150°) [31].

### Synthetic MP-containing wastewater

The model synthetic wastewater utilized in this study was prepared by spiking a known concentration of polyethylene into a mixture of distilled water and ethanol (9:1). Ethanol was added to reduce the surface tension of the mixture and increase the dispersion of the MP particles throughout the solution [32,33]. The mixture was magnetically stirred for 30 min prior to the removal study to further enhance MP dispersion, and stirred throughout the study to maintain good dispersion of MP particles in the water sample.

### Column-based removal

**Figure 1.**
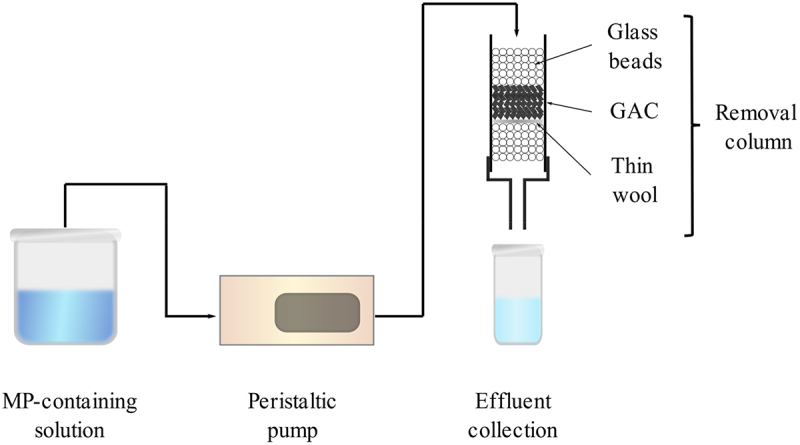
Schematic diagram of experimental setup.

MP removal study was conducted in a column as shown in the schematic diagram of the experimental setup shown in Figure 1.

The glass column (length = 20 cm, outer diameter = 15 mm) was filled with GAC as adsorbent/filter media at variable bed length and mass. The GAC was supported by glass beads (diameter = 5 mm) on top and bottom of the filter bed to facilitate the flow of water samples containing MP particles through the column. A very thin layer of wool was placed between the media and bottom glass beads to prevent the GAC being flushed into the effluent stream. Assuming that GAC particles are spherical, the bed porosity of GAC inside the column is 0.4240, calculated using Equation 1, where ε represents mean porosity, D represents column diameter (mm), and d refers to particle diameter (mm) [34]. The bed porosity in this study is well within the typical range of bed porosity (ε = 0.2595–0.4764) utilized in various chemical engineering operations e.g. separation and reaction processes [35].

(1) $$\;\varepsilon =0.39+1\frac{1.74}{{\left(\frac{\;D}{\;d}+1.14\right)}^{2}}$$

MP-containing wastewater was passed from the upper side of the column to the bottom gravitationally with the assistance of a peristaltic pump (Cole Parmer Easy-Load MasterFlex 7518-00) at a flow rate of 7 ml/min. Two parameters evaluated in this study are the MP concentration (0.2–1.0 g/L) and GAC bed length (7.5–15 cm). The MP removal process was conducted continuously for 2 h, and the effluent was visually observed to determine the breakthrough time. Each experiment was conducted in triplicates and the average values were reported.

MP removal performance was determined by the gravimetric method where the effluent collected was filtered using a filter paper (Smith 102 Qualitative), dried at room temperature, and weighed with an analytical balance. The difference in the mass of filter paper before and after filtration is considered as the mass of MP present in the effluent. Due to the possible presence of impurities in the distilled water utilized, an identical amount of distilled water was filtered and considered as a blank sample. The removal efficiency of the process was calculated using Equation 2.

(2) $$\text{Removalefficiency}\left(\%\right)=\frac{\text{MP}\text{in}\text{influent}\left(\text{g}\right)\;-\text{MP}\text{in}\text{effluent}\left(\text{g}\right)}{\text{MP}\text{in}\text{influent}\left(\text{g}\right)}\text{x}100\%$$

For quality control, several measures were taken throughout the experiment to prevent cross-contamination from the surroundings. The whole experiments were conducted in an enclosure, which can be accessed only by the hands of the laboratory personnel. The laboratory personnel have also worn pure cotton clothes and latex gloves to prevent fiber contamination from the worn attire. All experiments were conducted in triplicates, and the results are the average values obtained. All samples and related apparatus were covered with aluminum foil to reduce possible contamination with atmospheric MP.

## Results

### Characterization of GAC

**Figure 2.**
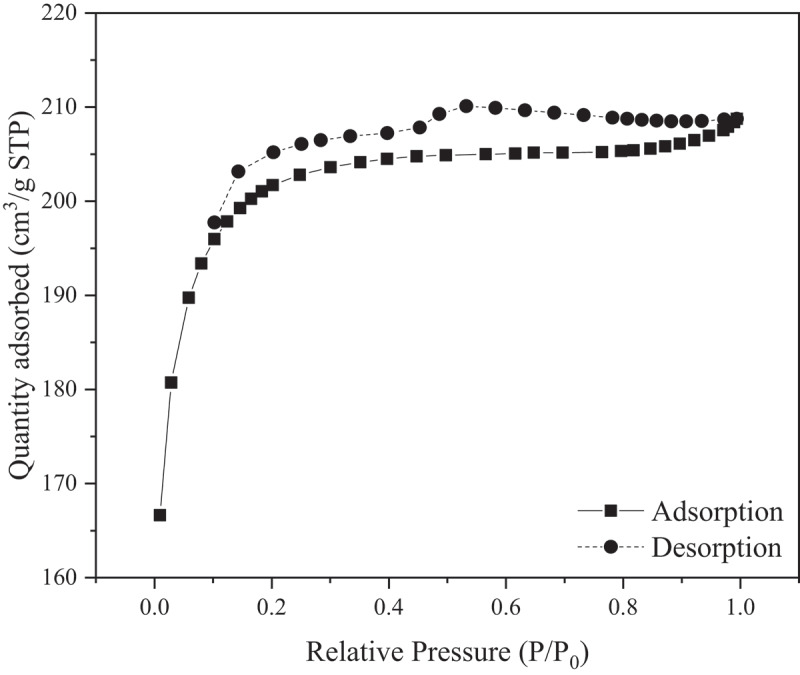
N_2_ adsorption–desorption isotherm of GAC media.

Nitrogen adsorption–desorption isotherms of the GAC used are shown in Figure 2. GAC exhibits a Type IV(a) isotherm with H4 hysteresis throughout the range of P/P_0_ tested, as shown in Figure 2. This represents a material of microporous–mesoporous type with characteristics of capillary condensation as indicated by the hysteresis [36]. The commercial GAC utilized in this study possesses a surface area of 682.24 m^2^/g, average pore width of 1.88 nm, and average pore volume of 0.032 cm^3^/g. Note that this implies that the pores of the GAC media are significantly smaller than the average particle size of the MP utilized in this study.

**Figure 3.**
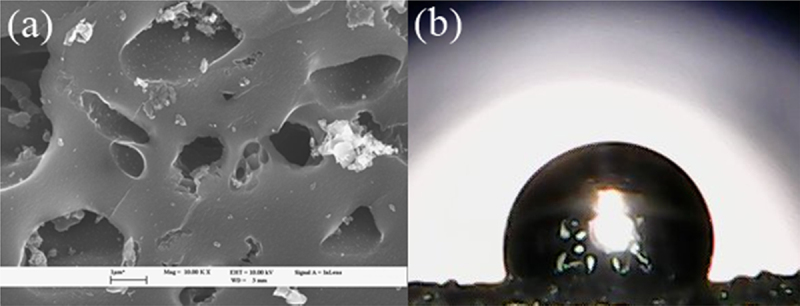
(a) Surface morphology of GAC and (b) contact angle measurement of GAC surface.

The surface morphology and the contact angle of the GAC surface are shown in Figure 3(a, b), respectively.

The surface morphology of GAC shown in Figure 3(a) at magnification of ×5000 indicates that the surface of the GAC is very rough and porous, in agreement with the findings from N_2_ adsorption–desorption isotherm analysis, which shows that GAC possesses pores in the microporous and mesoporous regions with abundance of the former. The contact angle measurement shown in Figure 3(b) shows that the water contact angle of the GAC surface is 108.8°, implying that the surface is slightly hydrophobic in nature [31].

### Effect of MP concentration

**Figure 4.**
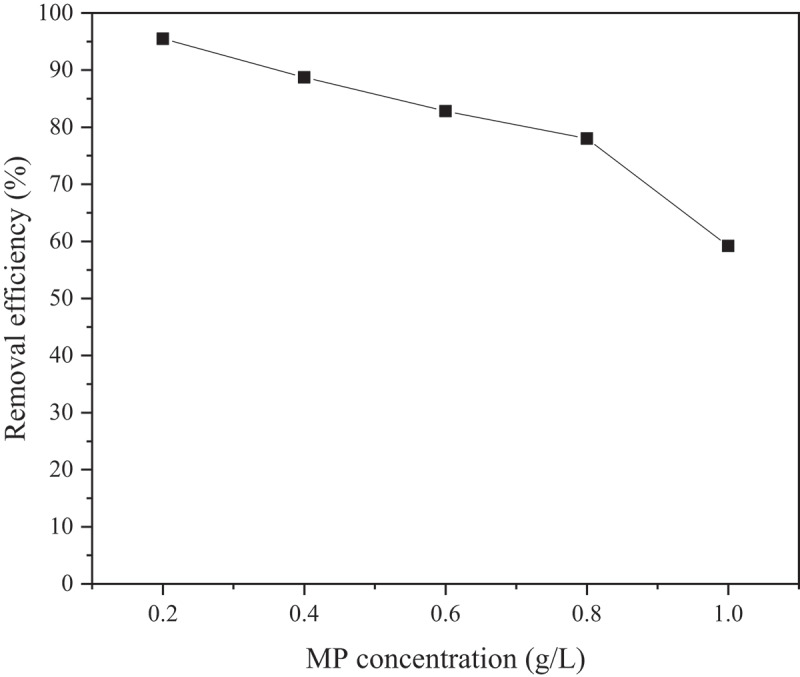
Removal efficiencies of MP at varying initial concentrations (0.2–1.0 g/L) using GAC with a fixed bed length of 7.5 cm.

The performance of MP removal at different initial concentrations (0.2–1.0 g/L) by a constant GAC bed length of 7.5 cm is shown in Figure 4 and summarized in Table 1.

**Table 1.** The performance of MP removal by GAC at different MP concentrations (0.2–1.0 g/L).

MP concentration (g/L)	MP in effluent (g)	Removal efficiency (%)
0.2	0.009	95.5 ± 0.6
0.4	0.045	88.5 ± 0.5
0.6	0.103	82.8 ± 0.7
0.8	0.176	78.0 ± 0.6
1.0	0.408	59.2 ± 0.8

As can be observed in Figure 4 and Table 1, the performance of GAC in eliminating MP decreases with an increase in MP concentration. MP removal obtained at the lowest MP concentration (0.2 g/L) is 95.5%, but this is immediately dropped to values below 90% with further increase in MP concentration and eventually to 59.2% with the highest MP concentration tested (1.0 g/L). As the GAC utilized in this study is in granular shape, the packing of the materials is less compact than smaller-sized media e.g. powder [37,38]. Having more space in the GAC bed results in the higher possibility of small-sized MPs passing through the available pathways. As the MP concentration increases, greater amounts of MP particles are able to pass through the GAC bed, leading to deterioration in the performance of MP removal. MP removal efficiency obtained in this study can be compared with other related column-based MP removal studies, summarized in Table 2.

**Table 2.** Comparison of MP removal efficiency of this study with previous column-based MP removal studies.

Media	MP	Removal (%)	Reference
Corn straw, hardwood biochar	Polystyrene	>95	[12]
Pine and spruce bark biochar	Polyethylene and fleece fiber	85.4–100	[25]
Zeolite, molecular sieve	Polyethylene and polyamide	>96	[26]
Quartz sand	Polystyrene	83–100	[39]
GAC	Polyethylene and polystyrene	86.9–99.9	[40]
GAC	Various MPs	92.9	[41]
GAC	Polyethylene	59.2–95.5	This study

Table 2 shows that the MP removal efficiency obtained in this study is comparable to other column-based MP removal work, although having slightly lower performance. It should be emphasized that the MP concentration utilized in this study is significantly higher than typical values in literature between 1 and 40 mg/L [33,42–44]. A higher working concentration was selected in this study by taking into account the predicted increase in the MP concentration in water bodies in the near future [7]. Based on the observed performance, GAC column-based removal can be considered suitable for removing MP particles up to 0.2 g/L, which is five times greater than the maximum concentration utilized in other studies. In addition, optimizing other process parameters e.g. GAC bed length may further enhance the removal efficiency of the process.

### Varying GAC bed length

**Figure 5.**
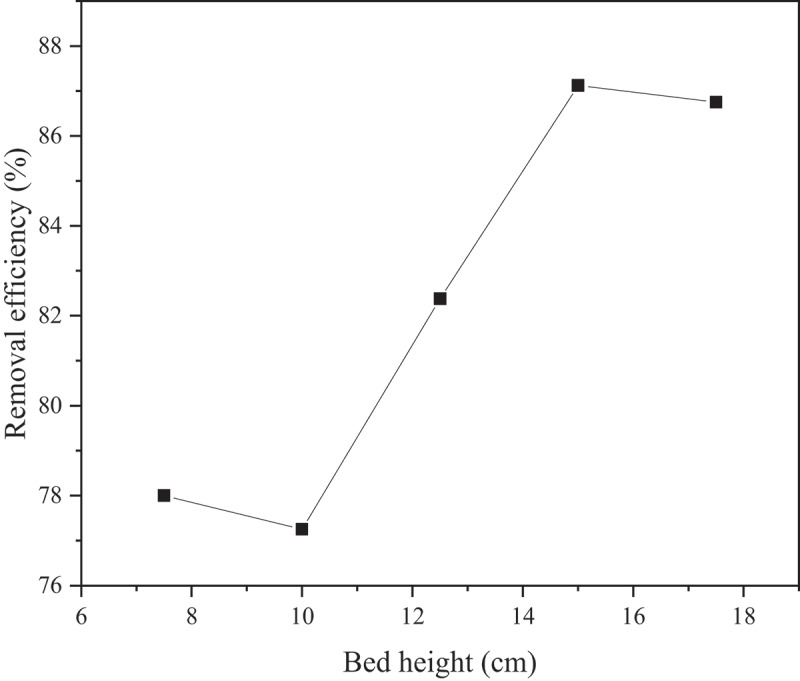
MP removal efficiencies (0.8 g/L) at varying GAC bed lengths (7.5–17.5 cm).

Based on the previous section, an MP concentration of 0.8 g/L was selected to further evaluate the possible enhancement of MP removal by varying the GAC bed length. The removal efficiencies of MP at different GAC bed lengths (7.5–17.5 cm) are shown in Figure 5 and summarized in Table 3.

**Table 3.** Removal efficiencies of 0.8 g/L MP by GAC of different bed lengths (7.5–17.5 cm).

GAC bed length (cm)	MP in effluent (g)	Removal efficiency (%)
7.5	0.176	78.0 ± 0.6
10	0.182	77.3 ± 0.2
12.5	0.141	82.4 ± 0.5
15	0.103	87.1 ± 0.4
17.5	0.118	86.7 ± 0.5

Initially, no significant change is observed by increasing the GAC bed length from 7.5 to 10 cm. Further increase of GAC bed length to 12.5 and 15 cm results in an enhancement of MP removal efficiencies up to 82.4% and 87.1%, respectively. The performance then slightly dropped to 86.7% with the highest GAC bed length of 17.5 cm; nonetheless, this is still higher than the other shorter GAC beds (7.5–12.5 cm). This implies that GAC is capable of retaining or adsorbing more MPs as the particles traveled through longer beds. The enhancement in the removal efficiency may be ascribed to the greater number of binding sites available and higher possible collisions between the MP and GAC particles with longer GAC bed length [43]. Based on these results, the process parameters should be optimized prior to conducting column-based MP removal as a slight variation of these parameters (MP concentration, bed length, etc.) could result in a significant reduction in the performance of the method in retaining MP particles.

## Discussion

As previously discussed, MP may be removed by column media through filtration, adsorption, or a combination of both processes. In this study, MP is conceivably immobilized and retained physically by the GAC media either by being (i) stuck between the GAC particles, (ii) entangled with small GAC particles or chips, (iii) trapped in the GAC porous structure, or (iv) adhered onto the GAC surface [12].

**Figure 6.**
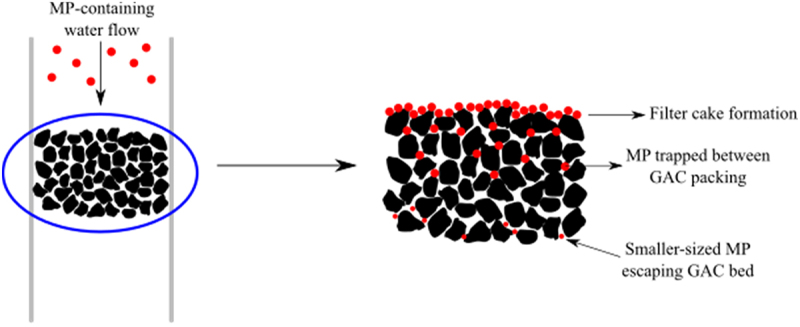
MP removal mechanism through the GAC column bed.

Based on experimental observation, it is presumable that as the MP-containing water was flowing through the GAC bed, most of the MP particles were filtered as they were unable to pass through the available pores/pathways between the GAC packing [37], as shown in Figure 6. Most of the MP particles are stuck on the upper layer of the GAC bed, eventually resulting in cake formation.

The cake layer can be removed through a backwashing process where water, air, or a mixture of water–air is passed through the column in a direction opposite to that used in the MP removal study. The collected backwashed water can be filtered where the MP particles can be recovered and subsequently recycled and reutilized or properly removed if the plastic particles are non-reusable. The fact that backwashing can facilitate the recovery of MP particles retained by GAC particles also makes this method more favorable.

In addition, the study on the effect of GAC bed length on MP removal is highly important toward scaling up the process for practical application. To obtain an optimized MP removal efficiency and a high recovery of MP, bed expansion between 25% and 30% is suggested [45,46]. Therefore, an ideal length of the column should be at least between 18.75 and 19.5 cm based on the optimal GAC bed length (15 cm) and the suggested bed expansion.

As previously shown, the surface morphology of GAC is very rough and porous. This facilitates the entanglement of MP particles inside the grooves on the GAC particle surface. This is similar to the result shown by Shen et al. [26], where MP particles were found to be trapped on the rough zeolite surface but not on the smoother surface of molecular sieve.

**Figure 7.**
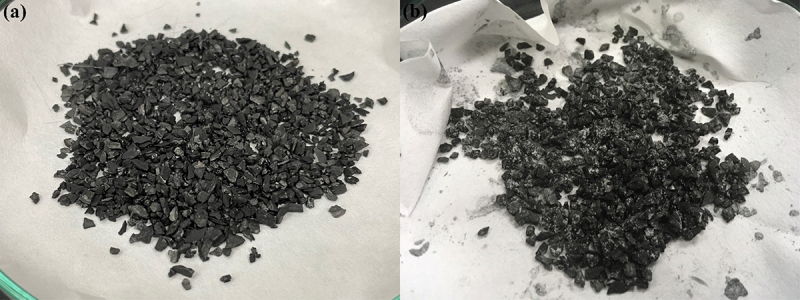
Physical conditions of GAC particles (a) before MP removal and (b) after MP removal.

Based on GAC characterization and cake formation, the removal process may be predominantly occurring by the first two mechanisms. The size of MP utilized in this study is also significantly larger than the pore size of the GAC utilized in this study; thus, it is impossible for the MP particles to enter the pore structure and get trapped inside. The GAC surfaces are only slightly hydrophobic, indicating weak retention ability, and thus only small parts of MP are perceivably attached onto the GAC surface through adsorption [26]. This can be further supported by the physical conditions of the GAC particles after undergoing an MP removal process as shown in Figure 7 after taken out from the column in comparison to the GAC prior to the removal process.

Based on the figure, only a small amount of MP particles is visibly attached onto the surface of the GAC particles compared to the amount of MP forming cake at the top of the GAC bed surface. As filtration is assumed to be the main mechanism of MP removal in this column-based method while adsorption is expected to only contribute to a small part of the removal process, the study on adsorption isotherm is not applicable.

In the context of filtration, various filtration membranes have been reported to successfully eliminate MP particles from wastewater. However, most of the membrane filters are produced from plastic-based materials such as polycarbonate (PC), polytetrafluoroethylene (PTFE), polyvinylidene fluoride (PVDF), and cellulose acetate [47–49]. Prolonged usage of membrane filters made from these materials would result in abrasion of the membrane and consequently resulting in secondary pollution of plastic particles [47,50,51]. On the other hand, the utilization of GAC in a column-based removal can prevent the possibility of a secondary pollution, making the method more appealing and advantageous.

For further improvement of the process, necessary modifications should be employed to enhance the removal performance, especially at higher MP concentrations. The column porosity could be slightly reduced by utilizing smaller-sized GAC to enable higher retention of MP particles by the GAC particles. It should be emphasized that a reduction in column porosity would lead to higher head loss, which would be detrimental toward water flow inside the column; therefore, a balanced condition between these two parameters should be achieved [52,53]. In addition, the surface of the GAC particles may be modified with suitable surfactants, which would allow better interaction between the GAC and MP particles through electrostatic attraction or hydrophobic interaction [26].

## Conclusion

In this work, synthetic water samples containing high MP concentration were subjected to a column-based continuous flow removal using commercial GAC as adsorbent/filter media. The method performed quite well with the highest MP removal of 95.5% at 0.2 g/L MP. Both initial MP concentration and GAC bed length were found to be significant factors in affecting the MP removal efficiency. The performance was reduced significantly with higher MP concentrations up to 1.0 g/L as the GAC became unable to accommodate and immobilize higher amounts of MP particles. At constant MP concentration (0.8 g/L), the MP removal performance was significantly enhanced to 87.1% by increasing the GAC bed length. Even though high removal efficiency is attainable through this study, the result implies that various other process parameters and proper designing should be considered for a column-based MP removal to be utilized and upscaled as the properties of MP-containing water may differ from one place to another.

## Data Availability

The data that support the findings of this study are available from the corresponding author upon reasonable request.
